# Synthesis of Luminescent Graphene Quantum Dots with High Quantum Yield and Their Toxicity Study

**DOI:** 10.1371/journal.pone.0144906

**Published:** 2015-12-28

**Authors:** Dan Jiang, Yunping Chen, Na Li, Wen Li, Zhenguo Wang, Jingli Zhu, Hong Zhang, Bin Liu, Shan Xu

**Affiliations:** 1 School of Stomatology, Lanzhou University, Lanzhou, China; 2 State Key Laboratory for Oxo Synthesis and Selective, Lanzhou Institute of Chemical Physics, Lanzhou, China; 3 Department of Heavy Ion Radiation Medicine, Institute of Modern Physics, Chinese Academy of Sciences, Lanzhou, China; SPECS Surface Nano Analysis GmbH, GERMANY

## Abstract

High fluorescence quantum yield graphene quantum dots (GQDs) have showed up as a new generation for bioimaging. In this work, luminescent GQDs were prepared by an ameliorative photo-Fenton reaction and a subsequent hydrothermal process using graphene oxide sheets as the precursor. The as-prepared GQDs were nanomaterials with size ranging from 2.3 to 6.4 nm and emitted intense green luminescence in water. The fluorescence quantum yield was as high as 24.6% (excited at 340 nm) and the fluorescence was strongest at pH 7. Moreover, the influences of low-concentration (12.5, 25 μg/mL) GQDs on the morphology, viability, membrane integrity, internal cellular reactive oxygen species level and mortality of HeLa cells were relatively weak, and the *in vitro* imaging demonstrated GQDs were mainly in the cytoplasm region. More strikingly, zebrafish embryos were co-cultured with GQDs for *in vivo* imaging, and the results of heart rate test showed the intake of small amounts of GQDs brought little harm to the cardiovascular of zebrafish. GQDs with high quantum yield and strong photoluminescence show good biocompatibility, thus they show good promising for cell imaging, biolabeling and other biomedical applications.

## Introduction

Graphene quantum dots (GQDs), a kind of promising carbon-based luminescent materials, have drawn researchers attention to biomedicine fields owing to their excellent optical-electronic characteristics and superior biocompatibility. As a zero-dimensional carbon-based material, GQDs are planar nanomaterials with lateral dimension ranging from 2 to 20 nm [1–4], showing intrinsic luminescence as a result of quantum confinement, surface defects and edge structure [1, 5–7]. In addition, GQDs also exhibit single atom layered structure just as conventional graphene do [8, 9]. Furthermore, compared with semiconductor quantum dots, GQDs show many advantages such as chemical inertness, ease of fabrication and low toxicity [10]. Besides, GQDs can also greatly reduce the toxicity induced by the heavy metals in traditional quantum dots [11, 12]. Thus, many different kinds of applications of GQDs have been investigated, such as biosensing [13], photovoltaic devices [14], cellular imaging [15], drug delivery and photodynamic therapy [16, 17].

With unique characteristics and various applications, the preparation of GQDs has been highly concerned. Currently, numerous top-down and bottom-up preparation methods are the focus of attention to develop environment-friendly, low-toxicity substitutes for semiconductor quantum dots. For the bottom-up synthesis [18, 19], GQDs are usually produced by condensation of aromatic compounds in liquid-phase reaction. The method is time-consuming and is difficult for purification. In comparison, the top-down preparation methods mean the cutting of large graphene sheets into ultra-small graphene with lateral dimension ranging from 2 to 20 nm, which is comparatively simple and effective. This method includes hydrothermal process [20], electrochemical oxidation [21], chemical exfoliation and so on [22]. Nevertheless, the relative low quantum yields and difficulty in the precise control of the GQDs’ morphology constrain their mass scale preparation. Therefore, it is dramatically imperative to develop synthetic method to manufacture high-property GQDs in mass scale. Recently, C-C bonds of GO were broken and cut into tiny dots with an average lateral size of 40 nm through photo-Fenton reaction [8]. It is noteworthy that the product yield of GQDs was ∼45%. While, their photoluminescence (PL) intensity was too weak to be available for the naked eye and thus limiting bioimaging and other optical applications. It has been reported that highly blue-luminescent GQDs were produced through hydrothermal process of GO and ammonia solution and the highest PL quantum yield was 24.6% [23]. In the process, the GO was highly oxidised. According to aforementioned investigations, the product of photo-Fenton reaction can be used as the precursor of the hydrothermal experiment in order to combine their different advantages. However, to the best of our knowledge, no literature reports further treatment of the product of photo-Fenton reaction to increase the PL intensity. Thus, we develop an ameliorative photo-Fenton reaction followed by a simple hydrothermal process to prepare luminescent GQDs with higher PL quantum yield.

Moreover, GQDs display strong PL property, which is considerably helpful for bioimaging, biolabeling and other biomedical applications. However, the key criteria that are required for the biomedical applications of GQDs are their cytotoxicity. In contrast with micrometer-sized GO sheets, nano-sized GQDs could be internalized into cells faster and the cytotoxicity of GQDs was relatively lower [9]. Nitrogen-doped GQDs were co-cultured with HeLa cells and did not impose considerable toxicity on HeLa cells by 3-(4, 5-dimeth-ylthiazol-2-yl)-2, 5-diphenyl tetrazolium bromide test (MTT) assay [23], and same results were also obtained by MTT assay [24]. Previous research has shown GQDs prepared by oxidation approach exhibited no apparent *in vitro* and *in vivo* toxicity [25]. Moreover, GQDs with various functional groups (NH_2_, COOH, and CO-N (CH_3_)_2_, respectively) showed low cytotoxicity to A549 cells in spite of various chemical modification [26]. However, it should be mentioned that most of the existing cytotoxicity studies are based on MTT assay, and a systematical cytotoxicity evaluation is necessary for the biocompatibility assessment of GQDs. Besides, there are few reports investigating the cardiovascular developmental toxicity of GQDs. Recently, with low husbandry cost, fast propagation, high degree of homology to humans’ gene and easy acquisition, zebrafish has been proved to be an effective vertebrate model on the evaluation of nano-materials’ developmental toxicity, such as silver, gold, platinum nanoparticles and titanium dioxide nanoparticles [27, 28].

In this paper, high quantum yield GQDs were prepared by an ameliorative photo-Fenton reaction and a subsequent hydrothermal process. Transmission electron microscope (TEM), Atomic force microscope (AFM), X-ray photoelectron spectrometer (XPS), powder X-ray diffraction (XRD), and Fourier transform infrared (FTIR) spectroscope were employed to characterize the microstructures of as-prepared GQDs. UV-visible absorption and normalized PL spectra were applied to analyze the optical properties of GQDs. Furthermore, the cytotoxicity of GQDs was systematically evaluated by a series of assays using HeLa cells as the object of study. Then the zebrafish embryos were employed to investigate the cardiovascular developmental toxicity of GQDs.

## Experimental Section

### Synthesis of GQDs

The precursor GO was synthesized in accordance with the method described by Hummer with a modification in the reported work [29]. GQDs were synthesized by photo-Fenton reactions of GO and one-step hydrothermal method. In the synthesis process, GO solution (5 mL, 1 mg/mL) was first mixed carefully with hydrogen peroxide (20 mL, 200 mM) and FeCl_3_ solution (0.5 mL, 1 mM) in a quartz tube (40 mL) with vigorous stirring and the final pH of the solution was adjusted to 4 with HCl solution. Then the horizontally placed quartz tube filled with mixture was under a mercury lamp (365 nm, 1000 W, Bilon, Shanghai, China) for UV irradiation about 15 min. The reaction products were poured into a dialysis bag (retained molecular weight: 3000 Da) for 2 days to remove iron ions and other small moleculars. Then the 200 mL resulting solution, 6mL hydrogen peroxide (30%) and 2 mL ammonia (25–28%) were then transferred into a 250 mL Teflon-lined stainless-steel autoclave and heated at 180°C for 5 h. The reaction mixture was filtered through a 0.22 μm microporous membrane to separate the larger sized substance and a brown filtrate was obtained. Then the filtrate was transfered into a dialysis tube (retained molecular weight: 1000 Da) for 2 days to remove trace ammonia and other small molecular. The pH of the final GQDs solution was adjusted to 7. The purified GQDs displayed strong green fluorescence under ultraviolet excitation and the product yield (m(GQDs)/m(GO). m(GO) can be obtained by the product of volume and concentration) was about 11.1 ± 0.9 wt%.

### Characterization

TEM (JEOL, JEM-2010) was applied to observe the morphology of the GQDs using an accelerating voltage of 200 kV. AFM image was obtained on a Multimode 8 atomic force microscope. XPS analyses were carried out on an X-ray photoelectron spectrometer (Physical Electronics, USA) with an Al-K Alpha as the X-ray source. FTIR spectrum was analyzed on a Fourier transform infrared spectrometer (Bruker IFS66V), and XRD analyses were investigated by X-ray diffraction patterns (Philips X’Pert Pro.). UV-vis absorption spectra of GQDs in water was analysed on a Hitachi U-3010 UV-vis spectrophotometer. PL characterization of GQDs solutions was obtained with an F-4500 fluorescence spectrophotometer.

### Cell cultures

HeLa cells (human cervical cancer cells, Shanghai Institutes of Biological Sciences) were cultured in RMPI-1640 medium (Hyclone) with 10% fetal bovine serum under a constant temperature of 37°C, 95% air and 5% CO_2_ humidified condition. The medium was refreshed every two days and the cells were harvested from the cultures by trypsin/EDTA solution and were re-suspended in fresh complete medium before plating.

### Cell morphology and imaging

HeLa cells at a density of 2×10^5^ per well were seeded into a 6-well plate and cultured for 24 h. After the cell medium was removed, cells were co-cultured with GQDs at concentration of 100 μg/mL for 24 h at 37°C. Cells cultured in the medium without GQDs were taken as the control. The cell morphology was observed under an optical microscopy. For cell imaging, HeLa cells were co-cultured with GQDs at concentration of 50 μg/mL for 24 h at 37°C. Successively, the cells were washed with PBS for three times to remove GQDs followed by fixing them in PBS with 4% paraformaldehyde for half an hour at room temperature. Finally, the resulting cells were observed with a laser scanning confocal microscopy (ZEISS LSM700, Germany) at the excitation of 405 nm laser beams.

### Cell counting kit-8 (CKK-8) assay

1×10^4^ HeLa cells were seeded in a 96-well plate for 24 h. Various concentration of 0, 12.5, 25, 50, 100, 200 μg/mL of GQDs were introduced into the cells for another 24 h. Then the cells were washed with PBS for three times. 100 μL of cell culture media and 10 μL of CCK-8 dye were added to each well and incubated for 2 h at 37°C. Medium and CCK-8 solution without cells were taken as the blank group. Plates were then analyzed with a microplate reader (BIO-RAD iMark, USA) at the wavelength of 490 nm. Five replicate wells were done for each concentration per plate. The whole assay was repeated for three times. Results were expressed as mean ± standard deviation (SD).

### LDH assay

The LDH assay is applied to evaluate the cell membrane integrity. Therefore, the cytotoxicity induced by GQDs was mearsured through the detection of the LDH level released in the supernatant by cells. Briefly, HeLa cells were seeded into plates as previously described methods. Then the cells were incubated with GQDs of different concentrations (0, 12.5, 25, 50, 100 and 200 μg/mL) for another 24 h. About the positive control treatment, 10 μL of lysis solution was added to the control well for 30 min before centrifugation (400 g × 5 min). After the centrifugation, 120 μL supernatant was transferred from each well to a fresh 96 well plate along with 60 μL LDH assay reagent (Beyotime Institute of Biotechnology, China) and incubated for 30 min in the dark at room temperature. The absorbance values were recorded by microplate reader at 490 nm.

### ROS assay

HeLa cells were seeded in 24-well plates at the density of 1×10^5^ cells per well. After 24 h incubation, HeLa cells were exposed with different concentrations (0, 12.5, 25, 50, 100 and 200 μg/mL) of GQDs for another 24 h. After the cells washed with PBS for twice, serum-free medium with 2, 7-dichlorofluorescein diacetate (H_2_DCF-DA) solution (10 μM) was added to each well and incubated them in the dark for 30 min at 37°C. Finally, extra H_2_DCF-DA was removed by washing each well with PBS, and the fluorescence intensity was measured by microplate reader under 490 nm excitation wavelength.

### Annexin-V apoptosis assay

HeLa cells were seeded in 6-well plates at the density of 1×10^5^ cells per well. After 24 h incubation, medium was substitued by fresh medium containing GQDs at various concentrations (0, 12.5, 25, 50, 100 and 200 μg/mL), separately. Cells were then trypsinized, washed twice with PBS and re-suspended in 500 μL binding buffer. Then 5 μL of FITC-conjugated Annexin V (Annexin V-FITC) and 5 μL of propidium iodide (PI) were added and incubated them for 15 min at room temperature in the dark. The stained cells were analyzed by flow cytometer (BD FACS Canto).

### Zebrafish maintenance and ethics statement

AB strains of wild-type zebrafish were maintained in a circulating aquarium system with embryonic medium (5 mM NaCl, 0.17 mM KCl, 0.33 mM CaCl_2_, 0.33 mM MgSO_4_, and 10–15% Methylene Blue (Sigma-Aldrich)) at 28 ± 1°C and 80% humidity. Embryos were collected and incubated in embryo medium under the condition of 28.5°C and 80% humidity. All the zebrafish used in the experiment were performed in accordance with the criterion of the Humane Treatment of Laboratory Animals, which was regulated by the Ministry of Science and Technology of the People’s Republic of China (MOST) and were ratified by the Institutional Animal Care and Use Committee (IACUC) of Institute of Modern Physics, Chinese Academy of Sciences. The Regulations for the Administration of Affairs Concerning Experimental Animals (1988.11.1) is attached to Institute of Modern Physics, CAS. Any unexpected deaths of the zebrafish embryo and larva in the experiment were cryopreserved. Once finishing experiments, embryos or zebrafish were anaesthetised with 0.02% MS-222 and sacrificed with a bleach solution (sodium hypochlorite 6.15%). Both zebrafish died in the experiment and the ones sacrificed after the experiment were sent to the administrator of zebrafish experimental center for further treatment.

### Cardiovascular toxicity in zebrafish and in vivo imaging

Fertilized embryos were co-cultured with control (embryo culture media, E3 media) and GQDs (12.5, 25, 50, 100 and 200 μg/mL) from 4 to 120 hour post fertilization (hpf). After exposure to control (E3 media) and GQDs (12.5, 25, 50, 100 and 200 μg/mL) for 96 hpf, ten randomly selected larvae of each treatment were washed with PBS for three times followed by fixing them in PBS with 4% paraformaldehyde at 4°C overnight. Then histological slices were prepared and stained with Hematein Eosin. Laser scanning confocal microscope (ZEISS, LSM700, Germany) was applied to observe the distribution of GQDs in the region of cardiovascular.

About the heart rate test, ten randomly selected zebrafish were recorded at 48, 72, 96, 120 hpf. Zebrafish larvae were anaesthetized using 0.01% MS-222 (Sigma, USA), and the heart beats (1 min) were observed by a stereoscopic dissecting microscope (Motic, SMZ-161, Motic China Group CO., LT, China) and Media Cruiser recording software (Canopus Corporation, Kobe, Japan). The data was assessed using EthoVision Heartbeat Detector software (Noldus Information Technology, Wageningen, Netherlands).

### Statistical analysis

The values were expressed as the mean with standard deviation (mean ± SD). Significance between groups has been analyzed using Student’s t-test. * indicates a statistical significance (*≤0.05) vs the control.

## Results and Discussions

### Synthesis and characterization of GQDs

**Fig 1:**
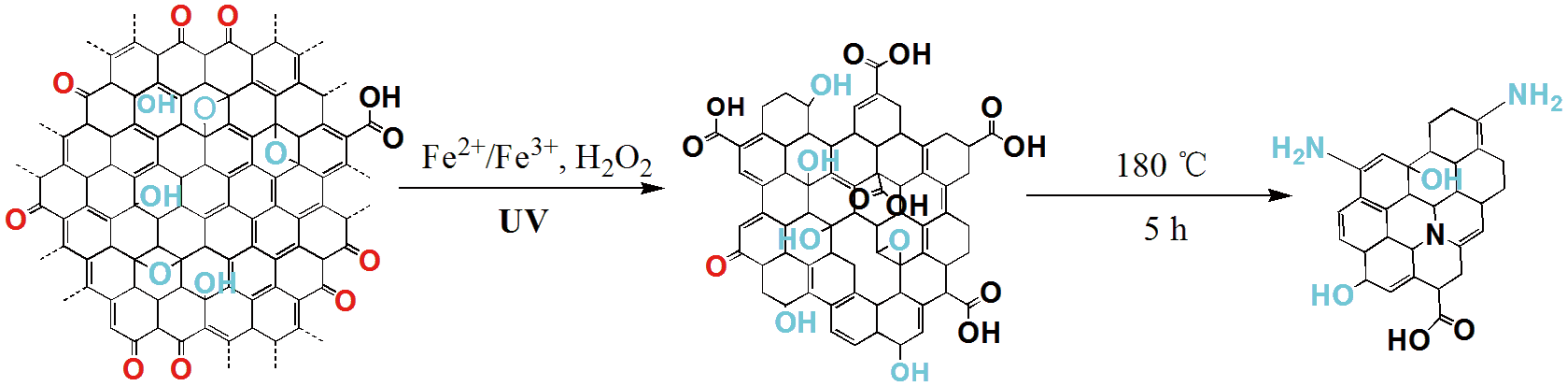
Schematic illustration for the preparation of GQDs.

Fig 1 illustrates the schematic process for the synthesis of GQDs. Step 1 is the photo-Fenton reaction of GO [8], which can effectively cut all GO sheets into tiny sheets and dots (Figure A in S1 File). Step 2 is a hydrothermal process for 5 h at 180°C with ammonia and hydrogen peroxide. After purification, the slightly yellow solution was freeze-dried and yellow powder (GQDs) was obtained. The synthetic process is low cost and relatively simple without the help of strong acid to cut large graphene oxide. Also, the synthesis process was very cheap in aqueous solution and the product yield is about 11.1 ± 0.9% in weight, higher than the other reported synthesis [23].

**Fig 2:**
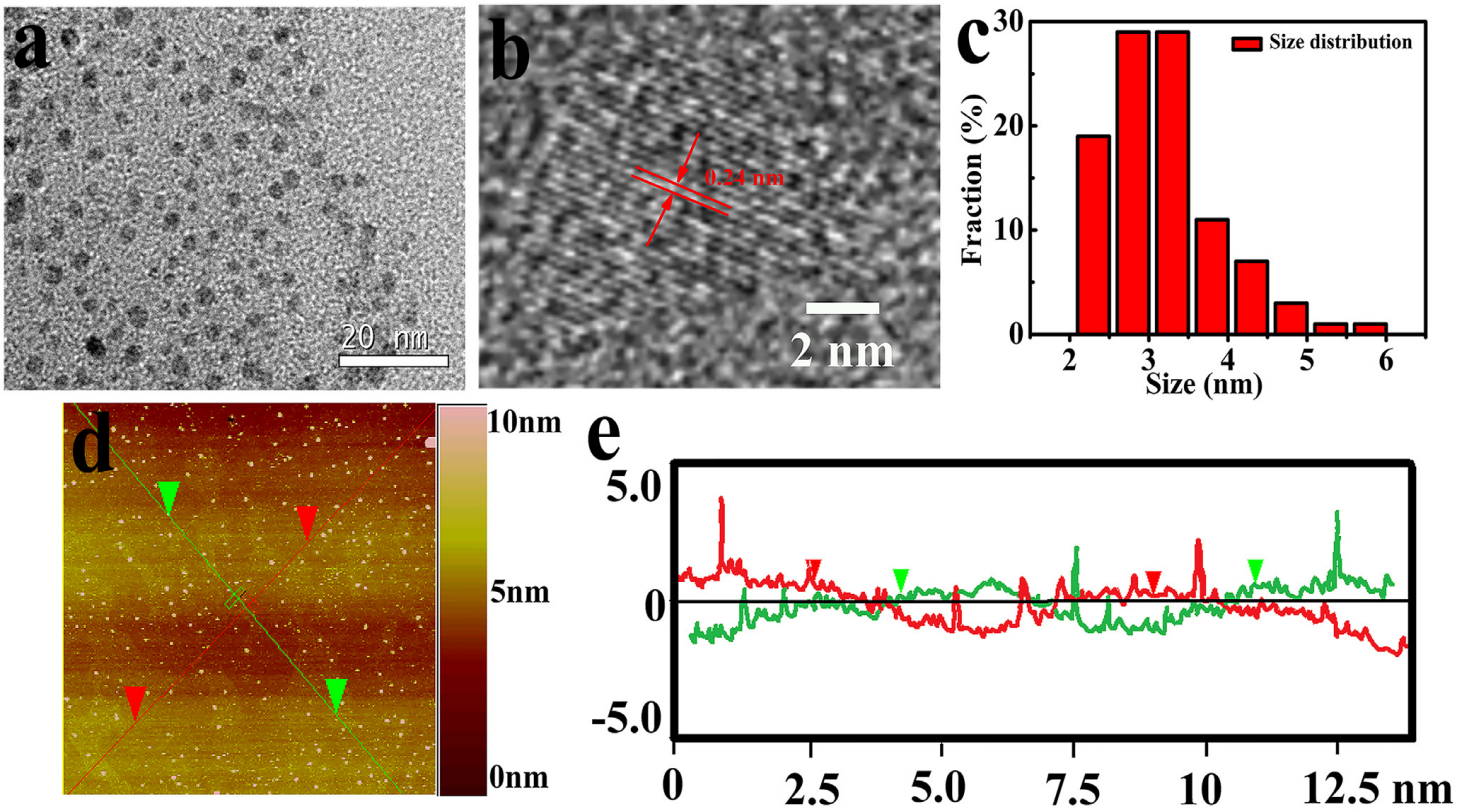
(a) TEM image of GQDs, (b) high-resolution TEM image of an individual GQD, (c) the size distribution of GQDs, (d) AFM image of GQDs and (e) the height profile along the lines in (d).

Fig 2a displays a typical TEM image of as-prepared GQDs, showing a relatively identical size distribution between 2.3 and 6.4 nm and the average lateral dimension is about 3.4 nm according to the statistical calculation of more than 200 dots (Fig 2c). High-resolution TEM image of a single GQD is exhibited in Fig 2b. The obvious crystal lattice demonstrates high crystallinity of GQDs and the lattice parameter of 0.24 nm represents the (1120) lattice fringe of graphene [23, 30]. The height distributions of AFM image (Fig 2d and 2e) are 0.6−3.5 nm, indicating 1–3 graphene layers [31].

**Fig 3:**
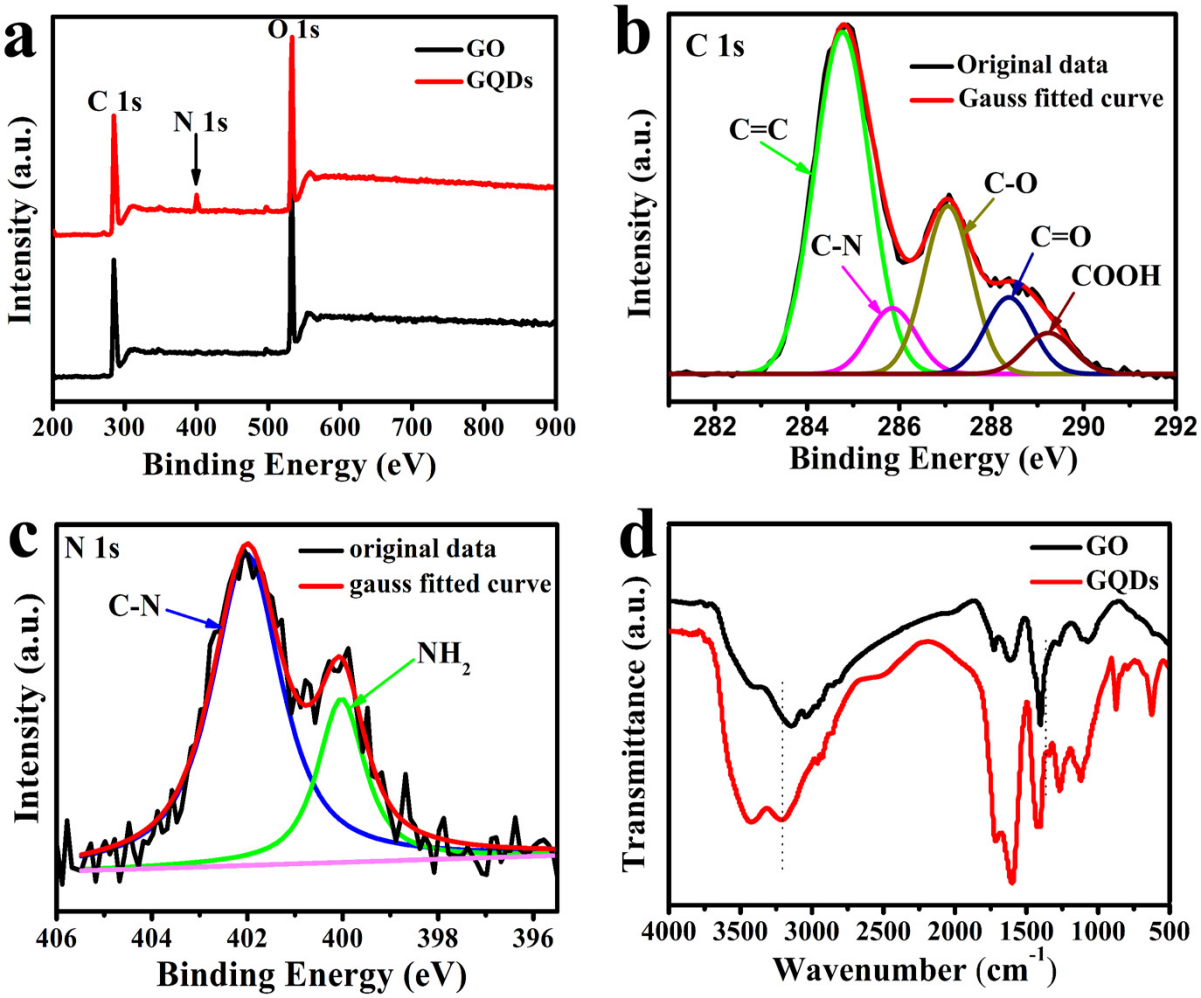
(a) XPS survey spectra and (b) well-fitted C 1s XPS spectra and (c) well-fitted N 1s XPS spectra of GQDs (d) FTIR spectra of GQDs.

XPS was applied to characterize the product in order to confirm the formation of GQDs. As depicted in Fig 3a, XPS survey spectra of the precursor (GO) and the as-prepared GQDs showed a distinct graphitic C 1s peak at ca. 285.4 eV and an O 1s peak at ca. 532.8 eV. The O/C atomic ratios in GQDs was 45.97% while GO was 39.22%, indicating that the oxidation level of GQDs was higher than that of GO. Compared with GO, a weak N 1s peak at 399.6 eV was observed in survey spectra of GQDs. This demonstrated that, through hydrothermal treatment of tiny GO dots with ammonia, N atoms were successfully introduced into the GQDs. The high resolution C 1s spectrum of the GQDs (Fig 3b) suggested the existence of the C–C/C = C (284.6 eV), C–O (287.1 eV), C = O (287.9 eV), COOH (289.0 eV) and C–N bond (286.1 eV) [31, 32], and the presence of oxygen functionalized groups leads to the hydrophilic nature of GQDs. The high resolution N 1s spectrum of the GQDs (Fig 3c) demonstrated the existence of both amide (399.8 eV) and C-N (402.0 eV), further indicated the successful introduction of N atoms into the GQDs, thus improving the hydrophilicity of GQDs. As shown in Fig 3d, the stretching vibrations of C−OH (3435 cm^-1^), C = O (1721 cm^-1^), C = C (1620 cm^-1^), C–H (1395 cm^-1^) and C–O (1260 cm^-1^) [19, 33] were observed in the Fourier transform infrared (FTIR) spectrum of GO and GQDs. In comparison, the stretching vibrations of oxygen-related groups in GQDs were stronger than that of GO. Moreover, the new peak at 1356 cm^-1^ was due to the C–N absorption bands [23, 34] and 3200 cm^-1^ was associated with the N–H stretching vibration of amine groups [32], once again demonstrating the successful incorporation of nitrogen atoms into the GQDs. The XRD results of GO sheets and GQDs were shown in Figure B in S1 File. The GO sample displayed a strong (002) diffraction peak at ca. 10.4°. Similar to other process [19, 35], the XRD of as-prepared GQDs showed a typical XRD profile of the GQDs centered at ca. 26°, indicating the interlayer spacing was 0.36 nm, due to the incorporation of oxygenated groups along the edges of the GQDs [23].

**Fig 4:**
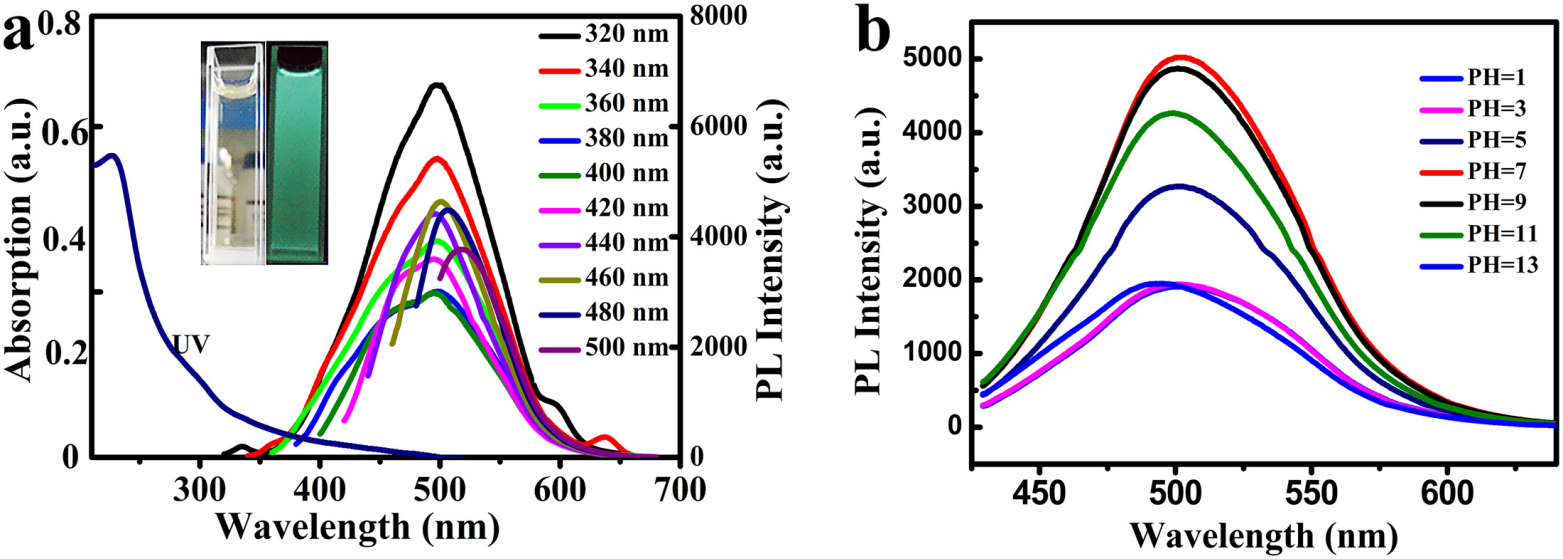
**a) UV-vis and PL spectra of the GQDs dispersed in water.** Inset is the photographs of the GQDs aqueous solution under visible light and 365 nm UV lamp, respectively b) The fluorescence spectra of GQDs in aqueous solutions at different pH values. The spectra are measured in PBS solutions and the excitation wavelength used is 400 nm.

The PL property of as-prepared GQDs was measured as well. The UV-visible absorption and PL spectra of GQDs were shown in Fig 4a. From the UV-visible spectrum, an distinct absorption peak around 227 nm was observed due to the π→π* vibration of aromatic groups [32, 36] and a long tail extended into the visible range.

When the GQDs were excited by a 365 nm UV lamp, the GQDs emitted green luminescence, which were bright enough to be observed by the naked eye and promising in applications of fluorescence imaging. However, it was noteworthy that the precursor of the GQDs showed weak and detectable PL in the ultraviolet region, but no fluorescence was detected from violet to visible light [8]. In addition, GQDs displayed excitation-dependent PL property. A red shift change was observed in the PL peak when the excitation wavelength changed from 320 to 500 nm. The PL quantum yields were measured according to the previous reported research [37]. when excited at 340 nm, as high as 24.6% of PL quantum yield was obtained (Table A in S1 File). The results were higher than most of the GQDs derived from GO, when compared with other reports,

For the effective introduce of semiconductor nanoparticles into the area of bio-imaging, it is critical to figure out the factors of biotic environment that affect the optical performance of GQDs. As displayed in Fig 4b, the fluorescence intensity of GQDs was extremely sensitive to pH. However, around the optimum pH value of 7 for biosome, greater fluorescence of GQDs was detected, which was better than CdSe/ZnSe/ZnS quantum dots with stronger fluorescence in aqueous alkaline medium and extremely sensitive to pH [38]. With high PL quantum yield and greater fluorescence at pH of 7, GQDs were appropriate for bioimaging.

### Cell morphology and distribution of GQDs

The morphology of HeLa cells after exposure with GQDs of 24 h were recorded to investigate the impact of GQDs on HeLa cells immediately. As shown in Figure C in S1 File, no obvious difference was observed between the GQDs treated cells (100 μg/mL) and the control cells. Moreover, few apoptosis cells were observed. Most cells adhered to the substrate firmly and were in normal spindle shape. These results suggested that GQDs had no obvious harmful effects on the morphology of HeLa cells.

**Fig 5:**
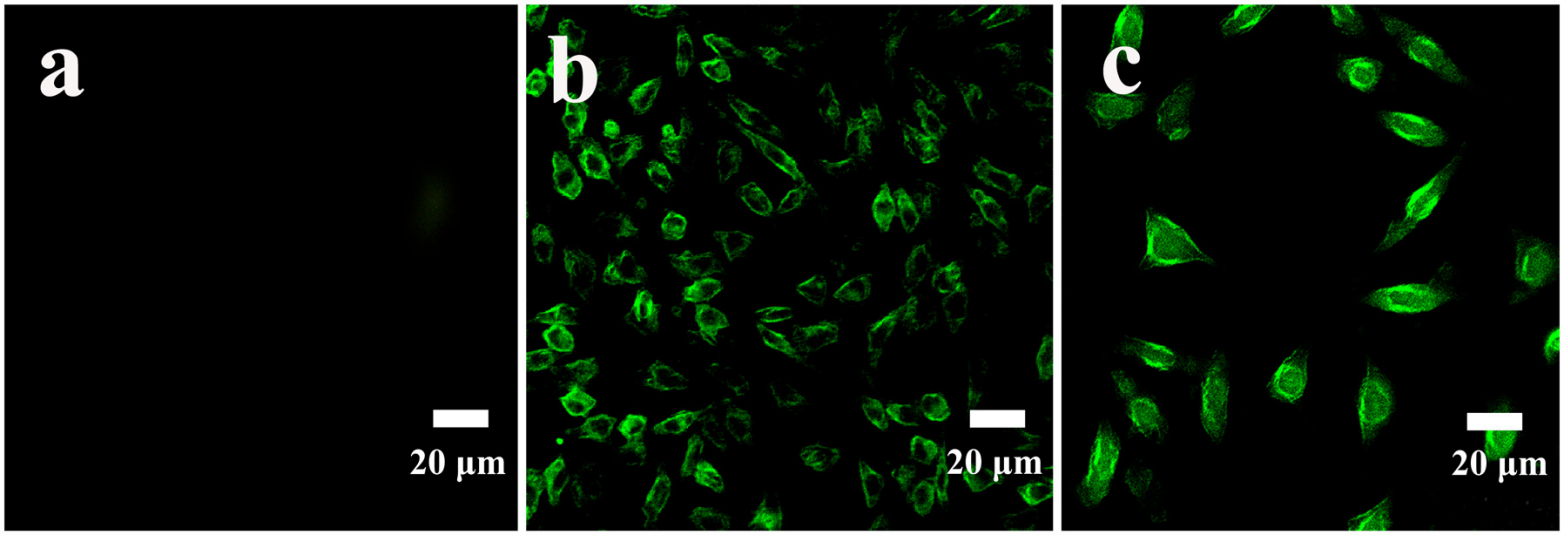
Fluorescence images of Hela cells: (a) control cells, (b) and (c) cells treated with 50 μg/mL of GQDs for 24 h at magnification of 10 and 20 times respectively.

In the study, the average lateral dimension of as-prepared GQDs was around 3.4 nm, which was quite small compared with HeLa cells. It has been reported that nano-sized GQDs could be internalized into cells faster compared with the micro-sized GO sheets [9]. Moreover, as-prepared GQDs emitted intense green luminescence, so it might be a good candidate for fluorescent probes in cell imaging. As shown in Fig 5, the laser scanning confocal microscopy image of the cells incubated (Fig 5b and 5c) with 50 μg/mL of GQDs was obviously brighter than that of the cells without GQDs treatment (Fig 5a), demonstrating that GQDs had internalized into the cells and mainly existed in cell membrane and cytoplasm region, in agreement with the previous literature reported by Yuan’s group [26]. The results revealed that even at the low concentration of 50 μg/mL, the fluorescence of the cells was still bright, which held great potential for bioimaging. In addition, GQDs could enter the cell cytoplasm, indicating another prospect of GQDs in drug delivery.

### Cytotoxicity of GQDs

**Fig 6:**
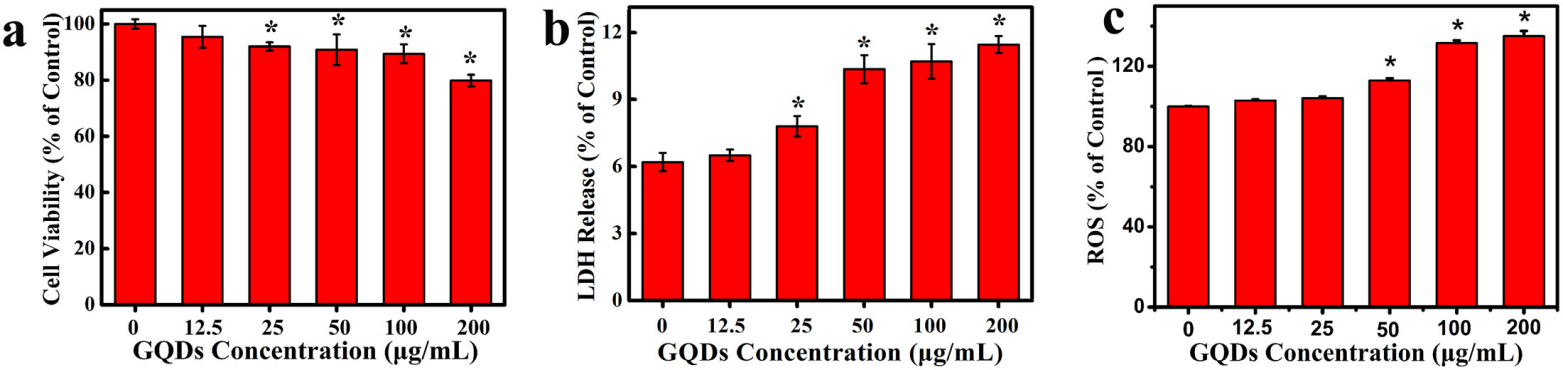
a) Cell viability, b) LDH release and c) internal cellular ROS level of Hela cells incubated with various concentrations of GQDs for 24 h. Data are presented as mean ± SD (n = 5 per group). (*≤ 0.05 between test and the control concentrations.)

Given the diverse inspiring biomedical applications of GQDs, such as drug delivery, protein analysis and bioimaging, it is critically important to evaluate the potential cytotoxicity. CCK-8 assay is an effective technique for the evaluation of cell viability [39]. As shown in Fig 6a, the cell viability decreased with the increase of GQDs concentration, but did not dramatically decrease. At the concentration ranging from 12.5 to 50 μg/mL, more than 90% cell viability was obtained. Even at the highest concentration of 200 μg/mL, nearly 80% cell viability was observed. These results revealed that a low dose of GQDs had well bio-compatibility and low toxicity to HeLa cells. GQDs also displayed little cytotoxicity to MC3T3 cells and MG-63 cells [40].

Cytotoxicity of GQDs can be further confirmed by Lactate dehydrogenase release (LDH) assay. Cell membrane integrity could be reflected by the LDH level out of cells [41]. The HeLa cells were exposed to a series of GQDs concentration for 24 h and then the release of extracellular LDH was measured. The results showed that LDH release was dose-related (Fig 6b). It also revealed that the LDH release levels at a series low concentration (12.5 and 25 μg/mL) were slightly higher than the control, indicating that a tiny fraction of cell membrane integrity of HeLa cells was compromised by GQDs. However, when exposed to high concentration of 200 μg/mL, the LDH level increased almost 50% compared with the control group, which might be speculated to be the reason that GQDs could contact with cell membrane and then cause the corresponding physical membrane damage. According to the literature, it has been reported that the GQDs can enter into the cells through endocytosis [42, 43]. These results suggested that a small dose of GQDs showed relatively lower toxicity, while high concentration (200 μg/mL) of GQDs displayed higher toxicity.

Reactive oxygen species (ROS) assay is an indicator for the detection of oxidative stress level in cells. A variety of studies demonstrated that some nanoparticles cause ROS generation on account of their chemical composition and surface characteristics [9, 41]. Thus, the free radicals and defects at the edge of GQDs suggest their potential for singlet oxygen generation [44]. As shown in Fig 6c, the level of ROS generation in HeLa cells caused by GQDs was dose-dependently. At the concentration ranging from 0 to 50 μg/mL, GQDs induced lower intracellular ROS level, while GQDs with higher concentrations induced more intracellular ROS. At the concentration of 200 μg/mL, the ROS level showed a significant increase in contrast with the control cells. It was possible due to the surface modification from GO to GQDs during the hydrothermal process [45].

**Fig 7:**
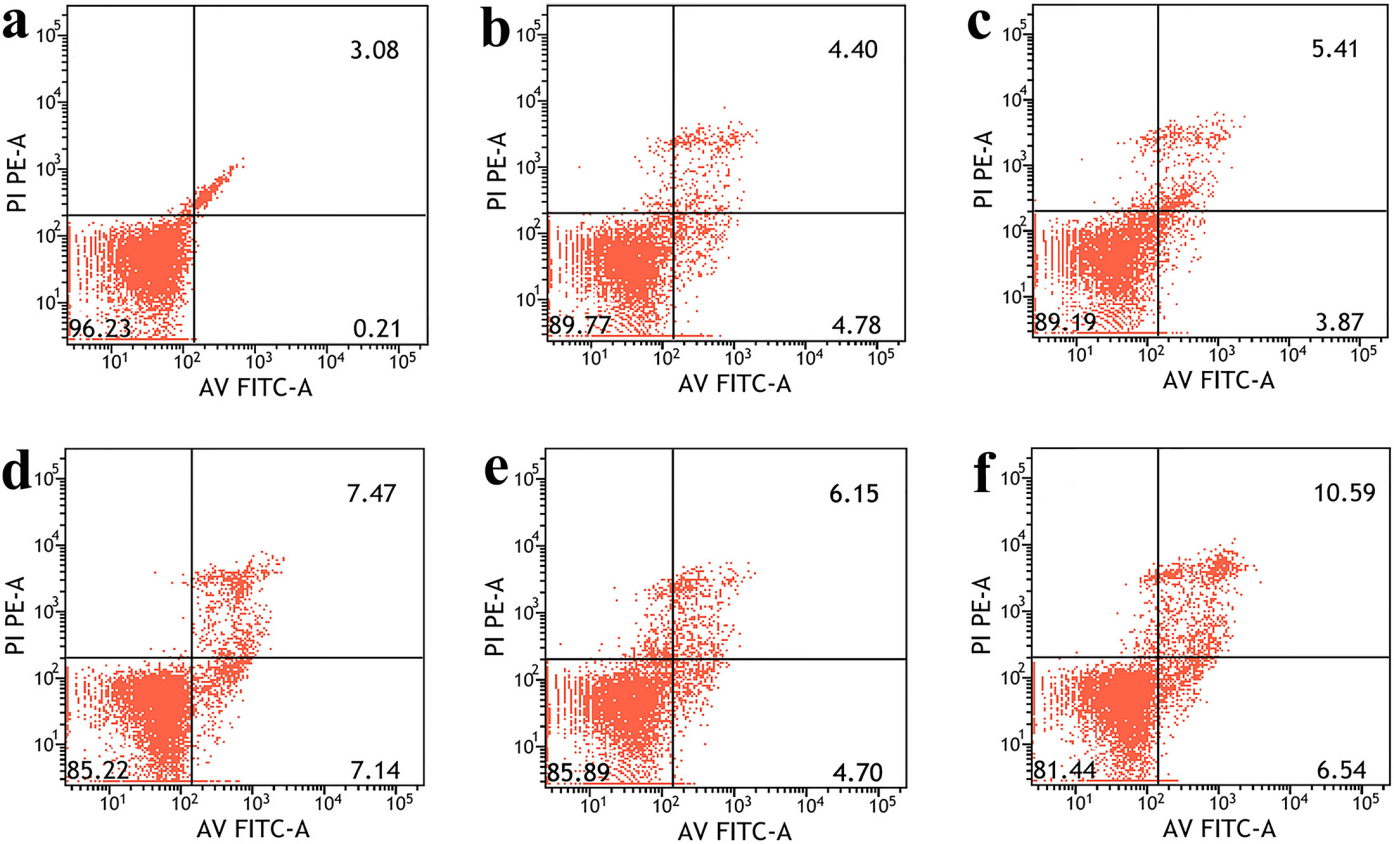
FACS results of the Annexin V-FITC and PI assay. Scatter diagrams of cells exposed to different concentrations of GQDs for 24 h.

Concerning the measurement of GQDs-induced cell apoptosis, HeLa cells were co-cultured with different concentrations of GQDs, and the evaluation of cell apoptosis was performed by flow cytometry. As shown in Fig 7, the dot plots showed that GQDs had induced a very small amount of apoptotic cells at the concentration of 12.5 and 25 μg/mL. Moreover, even at the concentration of 200 μg/mL, more than 80% of cells were in the third quadrant. These results indicated the apoptosis and necrosis rate induced by low concentrations of GQDs was negligible.

### Cardiovascular effects of GQDs on zebrafish embryos

**Fig 8:**
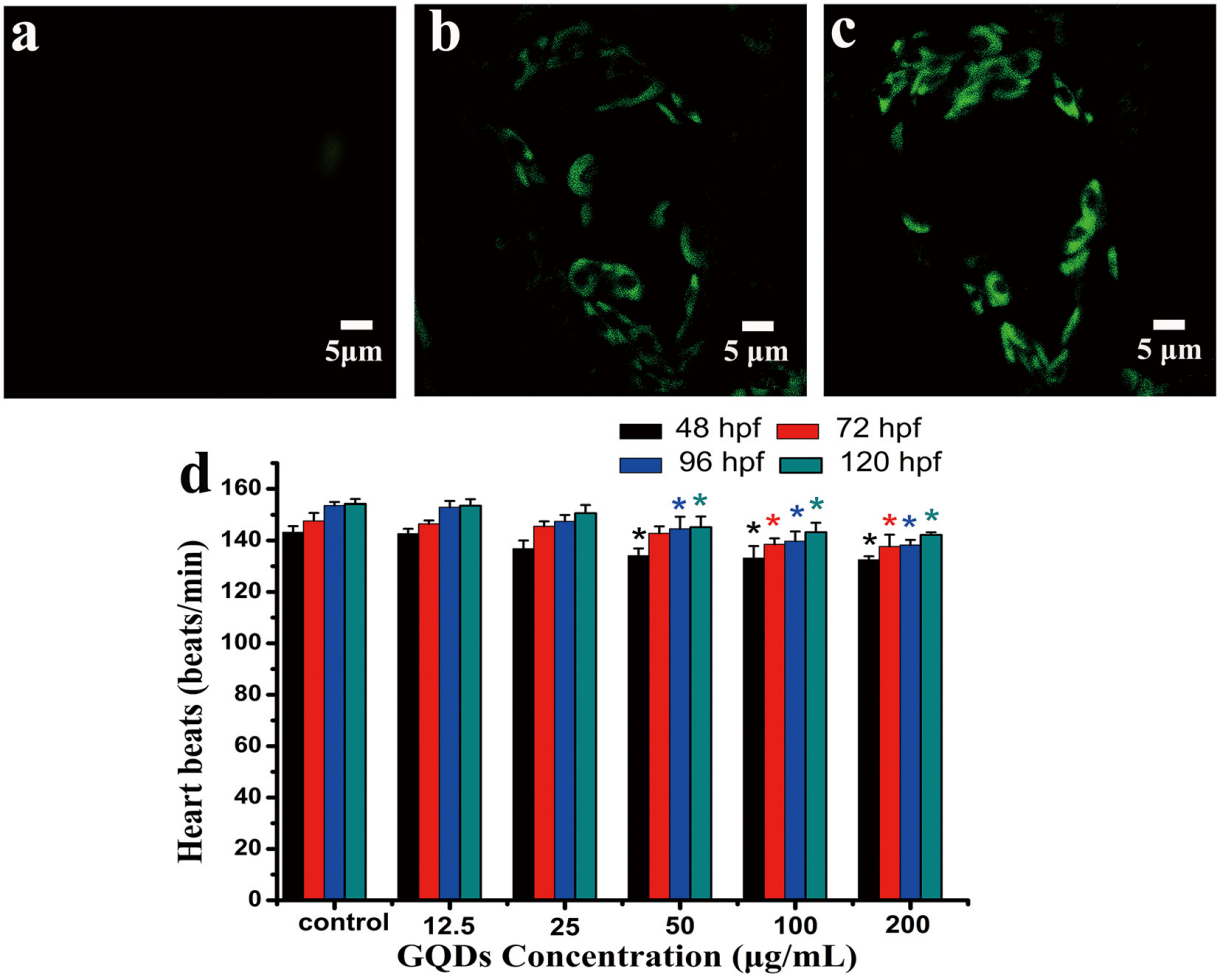
Confocal fluorescence images of histopathological slices in heart region at excitation of 405 laser beams and heart rate of zebrafish: a) control, b) larvae treated with 100 μg/mL, c) larvae treated with 200 μg/mL. d) heart rate of zebrafish embryos exposed to QGDs at 48, 72, 96 and 120 hpf. * denotes P < 0.05 compared with control. Values represent the mean ± SE.

After exposure to GQDs for 96 hpf, the GQDs biodistribution in cardiovascular of zebrafish was observed by LSCM. As shown in Fig 8a–8c, at the concentration of 100 and 200 μg/mL, the histological slices showed only myocardial cells were bright green compared with the control group and they were in typical spindle shape. It could also be seen clearly that GQDs were mainly in cytoplasm and not in cell nucleus region. The result was similar to the cell imaging displayed in Fig 5, indicating that GQDs would be a good alternative for imaging and drug delivery in cytoplasm.

Zebrafish has been proved to be an effective model animal on the evaluation of nanoparticles’ toxicity. Considering the distribution of GQDs was obviously observed in the heart region, after exposure to different concentration of GQDs, the heartbeats of zebrafish at 48, 72, 96, and 120 hour post fertilisation (hpf) were measured. As exhibited in Fig 8d, at a different time point, all of the heartbeats decreased dose-dependently. At low concentrations of 12.5 and 25 μg/mL, the heartbeats showed little difference compared with the control groups, indicating that the exposure of low concentration of GQDs may have little effects on the development of heart region of zebrafish embryos and larvae.

## Conclusions

GQDs were successfully fabricated by ameliorative photo-Fenton reaction and hydrothermal process. As-prepared GQDs emitted intense green luminescence under ultraviolet excitation and the fluorescence quantum yield was as high as 24.6%. Moreover, the weight yield of the GQDs was about 11.1 ± 0.9%. What’s more, GQDs had penetrated into the HeLa cells and mainly in the cytoplasm region. The cytotoxicity of GQDs was evaluated by CKK-8, LDH, ROS and Annexin-V apoptosis assays. Low-dose of GQDs (12.5 and 25 μg/mL) internalization did not induce significant cytotoxicity as proved by the high cell viability, low LDH release amount, low internal cellular ROS level and negligible amount of cell apoptosis. The in vivo test further demonstrated that low-dose of GQDs had little influences on zebrafish embryos and larvae. Thus, as-prepared GQDs with strong PL and good biocompatibility had great potential in biological and medical applications.

## Supporting Information

S1 File TEM image of tiny GO sheets and dots. Tiny GO sheets were prepared through photo-Fenton reaction of GO (Figure A). XRD patterns of GO sheets and GQDs (Figure B). Quantum yield of GQDs using quinine sulfate as a reference. Quinine sulfate in 0.1 M H_2_SO_4_ (QY = 0.54) was chosen as a standard with GQDs. The quantum yields of GQDs (in water) were calculated according to the formula: Φ = Φs(I/Is)(A/As)(ns/n). Where Φ is the quantum yield, I is the measured integrated emission intensity, n is the refractive index of the solvent (1.33 for water), and A is the optical density. The subscript “s” refers to the reference standard with known quantum yield. To minimize reabsorption effects, absorbencies in the 10 mm fluorescence cuvette were kept under 0.1 at 340 nm (Table A). Optical microscopy images: a) the control, b) GQDs-treated cells. HeLa cells optical microscopy images were recorded after treated with 0 and 100 μg/mL GQDs for 24 h (Figure C).
(DOCX)

## References

[1] Ponomarenko LA, Schedin F, Katsnelson MI, Yang R, Hill EW, Novoselov KS, et al Chaotic dirac billiard in grapheme quantum dots. Science. 2008; 320(5874): 356–358. 10.1126/science.1154663 18420930

[2] Sun YQ, Wang SQ, Li C, Luo PH, Tao L, Wei Y, et al Large scale preparation of graphene quantum dots from graphite with tunable fluorescence properties. Phys Chem Chem Phys. 2013; 15(24): 9907–9913. 10.1039/c3cp50691f 23673490

[3] Pan DY, Guo L, Zhang JC, Xi C, Xue Q, Huang H, et al Cutting sp^2^ clusters in graphene sheets into colloidal grapheme quantum dots with strong green fluorescence. J Mater Chem. 2012; 22(8): 3314–3318. 10.1039/c2jm16005f

[4] Xu F, Shi H, He X, Wang K, He D, Yan L, et al Masking agent-free and hannel-switch-mode simultaneous sensing of Fe3+ and Hg2+ using dual-excitation graphene quantum dots. Analyst. 2015; 140: 3925–3928. 10.1039/c5an00468c 25918855

[5] Liu Y, Gao B, Qiao Z, Hu Y, Zheng W, Zhang L, et al Gram-scale synthesis of graphene quantum dots from single carbon atoms growth via energetic material deflagration. Chem Mater. 2015; 27(12): 4319–4327. 10.1021/acs.chemmater.5b00774

[6] Zhu S, Tang S, Zhang J, Yang B. Control the size and surface chemistry of graphene for the rising fluorescent materials. Chem Commun. 2012; 48(38): 4527–4539. 10.1039/c2cc31201h 22473417

[7] Habiba K, Makarov VI, Avalos J, Guinel MJF, Weiner BR, Morell G. Luminescent graphene quantum dots fabricated by pulsed laser synthesis. Carbon. 2013; 64: 341–350. 10.1016/j.carbon.2013.07.084 27570249 PMC4999264

[8] Zhou XJ, Zhang Y, Wang C, Wu XC, Yang YQ, Zheng B, et al Photo-Fenton reaction of graphene oxide: a new strategy to prepare graphene quantum dots for DNA cleavage. ACS Nano. 2012; 6(8): 6592–6599. 10.1021/nn301629v 22813062

[9] Wu CY, Wang C, Han T, Zhou XJ, Guo SW, Zhang JY. Insight into the cellular internalization and cytotoxicity of graphene quantum dots. Adv Healthcare Mater. 2013; 2(12): 1613–1619. 10.1002/adhm.201300066 23703800

[10] Shen J, Zhu Y, Yang X, Li C. Graphene quantum dots: emergent nanolights for bioimaging, sensors, catalysis and photovoltaic devices. Chem Commun. 2012; 48(31): 3686–3699. 10.1039/c2cc00110a 22410424

[11] Derfus AM, Chan WCW, Bhatia SN. Probing the cytotoxicity of semiconductor quantum dots. Nano Lett. 2004; 4(1): 11–18. 10.1021/nl0347334 28890669 PMC5588688

[12] Lovric J, Cho SJ, Winnik FM, Maysinger D. Unmodified cadmium telluride quantum dots induce reactive oxygen species formation leading to multiple organelle damage and cell death. Chem Biol. 2005; 12(11): 1227–1234. 10.1016/j.chembiol.2005.09.008 16298302

[13] Ran X, Sun H, Pu F, Ren J, Qu X. Ag nanoparticle-decorated graphene quantum dots for label-free, rapid and sensitive detection of Ag^+^ and biothiols. Chem Commun. 2013; 49(11): 1079–1081. 10.1039/c2cc38403e 23282794

[14] Gupta V, Chaudhary N, Srivastava R, Sharma GD, Bhardwaj R, Chand S. Luminscent graphene quantum dots for organic photovoltaic devices. J Am Chem Soc. 2011; 133(26): 9960–9963. 10.1021/ja2036749 21650464

[15] Sun H, Ji H, Ju E, Guan Y, Ren J, Qu X. Synthesis of fluorinated and nonfluorinated graphene quantum dots through a new top-down strategy for long-time cellular imaging. Chem—Eur J. 2015; 21(9): 3791–3797. 10.1002/chem.201406345 25614445

[16] Ge J, Jia Q, Liu W, Guo L, Liu Q, Lan M, et al Red-Emissive Carbon Dots for Fluorescent, Photoacoustic, and Thermal Theranostics in Living Mice. Adv. Mater. 2015; 27(28): 4169–4177. 10.1002/adma.201500323 26045099

[17] Ge J, Lan M, Zhou B, Liu W, Guo L, Wang H, et al A graphene quantum dot photodynamic therapy agent with high singlet oxygen generation. Nat Commun. 2014; 5 10.1038/ncomms5596 PMC4143951 25105845

[18] Wang S, Chen Z, Cole I, Li Q. Structural evolution of graphene quantum dots during thermal decomposition of citric acid and the corresponding photoluminescence. Carbon. 2015; 82: 304–313. 10.1016/j.carbon.2014.10.075

[19] Wu X, Tian F, Wang WX, Chen J, Wu M, Zhao JX. Fabrication of highly fluorescent graphene quantum dots using L-glutamic acid for *in vitro*/*in vivo* imaging and sensing. J Mater Chem C. 2013; 1(31): 4676–4684. 10.1039/c3tc30820k 23997934 PMC3755467

[20] Qu D, Zheng M, Zhang L, Zhao H, Xie Z, Jing X, et al Formation mechanism and optimization of highly luminescent N-doped graphene quantum dots. Sci Rep. 2014; 4: 5294 10.1038/srep05294 24938871 PMC4061557

[21] Favaro M, Ferrighi L, Fazio G, Colazzo L, Di Valentin C, Durante C, et al Single and multiple doping in graphene quantum dots: unraveling the origin of selectivity in the oxygen reduction reaction. ACS Catal. 2015; 5(1): 129–144. 10.1021/cs501211h

[22] Liu F, Jang M-H, Ha HD, Kim J-H, Cho Y-H, Seo TS, et al Facile synthetic method for pristine graphene quantum dots and graphene oxide quantum dots: origin of blue and green luminescence. Adv Mater. 2013; 25: 3657–3662. 10.1002/adma.201300233 23712762

[23] Hu C, Liu Y, Yang Y, Cui J, Huang Z, Wang Y, et al One-step preparation of nitrogen-doped graphene quantum dots from oxidized debris of graphene oxide. J Mater Chem B. 2013; 1: 39–42. 10.1039/c2tb00189f 32260610

[24] Sun HJ, Wu L, Gao N, Ren J, Qu XG. Improvement of photoluminescence of graphene quantum dots with a biocompatible photochemical reduction pathway and its bioimaging application. ACS Appl Mater Interfaces. 2013; 5: 1174–1179. 10.1021/am3030849 23339586

[25] Chong Y, Ma Y, Shen H, Tu X, Zhou X, Xu J, et al The *in vitro* and *in vivo* toxicity of graphene quantum dots. Biomaterial. 2014; 35(19): 5041–5048. 10.1016/j.biomaterials.2014.03.021 24685264

[26] Yuan XC, Liu ZM, Guo ZY, Ji YH, Jin M, Wang XP. Cellular distribution and cytotoxicity of graphene quantum dots with different functional groups. Nanoscale Res Lett. 2014; 9: 108 10.1186/1556-276x-9-108 24597852 PMC3973856

[27] Asharani PV, Lianwu Y, Gong Z, Valiyaveettil S. Comparison of the toxicity of silver, gold and platinum nanoparticles in developing zebrafish embryos. Nanotoxicology. 2011; 5, 43–54. 10.3109/17435390.2010.489207 21417687

[28] Miao W, Zhu B, Xiao X, Li Y, Dirbaba NB, Zhou B, et al Effects of titanium dioxide nanoparticles on lead bioconcentration and toxicity on thyroid endocrine system and neuronal development in zebrafish larvae. Aquatic Toxicology. 2015; 161, 117–126. 10.1016/j.aquatox.2015.02.002 25703175

[29] Yan XB, Chen JT, Yang J, Xue QJ, Miele P. Fabrication of freestanding, electrochemically active, and biocompatible graphene oxide-polyaniline and graphene-polyaniline hybrid papers. ACS Appl Mater Interfaces. 2010; 2: 2521–2529. 10.1021/am100293r 20735069

[30] Peng J, Gao W, Gupta BK, Liu Z, Romero-Aburto R, Ge LH, et al Graphene quantum dots derived from carbon fibers. Nano Lett. 2012; 12(2): 844–849. 10.1021/nl2038979 22216895

[31] Feng YQ, Zhao JP, Yan XB, Tang FL, Xue QJ. Enhancement in the fluorescence of graphene quantum dots by hydrazine hydrate reduction. Carbon. 2014; 66:334–339. 10.1016/j.carbon.2013.09.008

[32] Jiang F, Chen DQ, Li RM, Wang YC, Zhang GQ, Li SM, et al Eco-friendly synthesis of size-controllable amine functionalized graphene quantum dots with antimycoplasma properties. Nanoscale. 2013; 5: 1137–1142. 10.1039/c2nr33191h 23282851

[33] Dong YQ, Pang HC, Ren SY, Chen CQ, Chi YW, Yu T. Etching single-wall carbon nanotubes into green and yellow single-layer graphene quantum dots. Carbon. 2013; 64: 245–251. 10.1016/j.carbon.2013.07.059

[34] Lai LF, Chen LW, Zhan D, Sun L, Liu JP, Lim SH, et al One-step synthesis of NH2-graphene from in situ graphene-oxide reduction and its improved electrochemical properties. Carbon. 2011; 49: 3250–3257. 10.1016/j.carbon.2011.03.05

[35] Tang LB, Ji RB, Cao XK, Lin JY, Jiang HX, Li XM, et al Deep ultraviolet photoluminescence of water-soluble self-passivated graphene quantum dots. ACS Nano. 2012; 6: 5102–5110. 10.1021/nn300760g 22559247

[36] Parimal R, Sandip D, Arnab S, Partha B, Pradip D, Arun KN. Graphene quantum dots from a facile sono-fenton reaction and its hybrid with a polythiophene graft copolymer toward photovoltaic application. ACS Appl Mater Interfaces. 2013; 5: 12672–12680. 10.1021/am4040174 24245528

[37] Sahu S, Behera B, Maiti TK, Mohapatra S. Simple one-step synthesis of highly luminescent carbon dots from orange juice: application as excellent bio-imaging agents. Chem Commun. 2012; 48(70): 8835–8837. 10.1039/c2cc33796g 22836910

[38] Liu Y-S, Sun Y, Vernier PT, Liang C-H, Chong SYC, Gundersen MA. pH-Sensitive Photoluminescence of CdSe/ZnSe/ZnS Quantum Dots in Human Ovarian Cancer Cells. J Phys Chem C. 2007; 111(7): 2872–2878. 10.1021/jp0654718 18985164 PMC2577287

[39] Yan L, Lin MM, Zeng C, Chen Z, Zhang S, Zhao XM, et al Electroactive and biocompatible hydroxyl-functionalized graphene by ball milling. J Mater Chem. 2012; 22: 8367–8371. 10.1039/c2jm30961k

[40] Zhu S, Zhang J, Tang S, Qiao C, Wang L, Wang H, et al Surface chemistry routes to modulate the photoluminescence of graphene quantum dots: from fluorescence mechanism to up-conversion bioimaging applications. Adv Funct Mater. 2012; 22: 4732–4740. 10.1002/adfm.201201499

[41] Gollavelli G, Ling YC. Multi-functional graphene as an *in vitro* and *in vivo* imaging probe. Biomaterials. 2012; 33: 2532–2545. 10.1016/j.biomaterials.2011.12.010 22206596

[42] Kang B, Chang S, Dai Y, Yu D, Chen D. Cell response to carbon nanotubes: size-dependent intracellular uptake mechanism and subcellular fate. Small. 2010; 6: 2362–2366. 10.1002/smll.201001260 20878638

[43] Zhang X, Li M, Wang YB, Cheng Y, Zheng YF, Xi TF, et al Cell response of nographene platelets to human osteoblast-like MG63 cells. J Biomed Mater Res Part A. 2014; 102A: 732–742. 10.1002/jbm.a.34751 23589384

[44] Markovic ZM, Ristic BZ, Arsikin KM, Klisic DG, Harhaji-Trajkovic LM, Todorovic-Markovic BM, et al Graphene quantum dots as autophagy-inducing photodynamic agents. Biomaterials. 2012; 33(29): 7084–7092. 10.1016/j.biomaterials.2012.06.060 22795854

[45] Singh SK, Singh MK, Kulkarni PP, Sonkar VK, Gracio JJ, Dash D. Amine- modified graphene: thrombo-protective safer alternative to graphene oxide for biomedical applications. ACS Nano. 2012; 6: 2731–2740. 10.1021/nn300172t 22376049

