# Supplementary material for: Synthesis of Luminescent Graphene Quantum Dots with High Quantum Yield and Their Toxicity Study
Source: PLoS One. 2015 Dec 28;10(12):e0144906. doi: 10.1371/journal.pone.0144906 (PMC4699207; doi:10.1371/journal.pone.0144906)
Supplement: S1 File — Tiny GO sheets were prepared through photo-Fenton reaction of GO (Figure A). XRD patterns of GO sheets and GQDs (Figure B). Quantum yield of GQDs using quinine sulfate as a reference. Quinine sulfate in 0.1 M H2SO4 (QY = 0.54) was chosen as a standard with GQDs. The quantum yields of GQDs (in water) were calculated according to the formula: Φ = Φs(I/Is)(A/As)(ns/n). Where Φ is the quantum yield, I is the measured integrated emission intensity, n is the refractive index of the solvent (1.33 for water), and A is the optical density. The subscript “s” refers to the reference standard with known quantum yield. To minimize reabsorption effects, absorbencies in the 10 mm fluorescence cuvette were kept under 0.1 at 340 nm (Table A). Optical microscopy images: a) the control, b) GQDs-treated cells. HeLa cells optical microscopy images were recorded after treated with 0 and 100 μg/mL GQDs for 24 h (Figure C). (DOCX) [file pone.0144906.s001.docx]

**Supporting Information**

**Synthesis of Luminescent Graphene Quantum Dots with High Quantum Yield and Their Toxicity Study**

Dan Jiang^1,2^, Yunping Chen^1,2^, Na Li^1^, Wen Li^1^, Zhenguo Wang^1,3^, Jingli Zhu^1^, Hong Zhang^3^, Bin Liu^1*^ and Shan Xu^2*^

^1^ School of Stomatology, Lanzhou University, Lanzhou, China

^2^ State Key Laboratory for Oxo Synthesis and Selective, Lanzhou Institute of Chemical Physics, Lanzhou, China

^3^ Department of Heavy Ion Radiation Medicine, Institute of Modern Physics, Chinese Academy of Sciences, Lanzhou, China

^*^ Corresponding author: liubkq@lzu.edu.cn, [xushan@licp.cas.cn](mailto:xushan@licp.cas.cn)


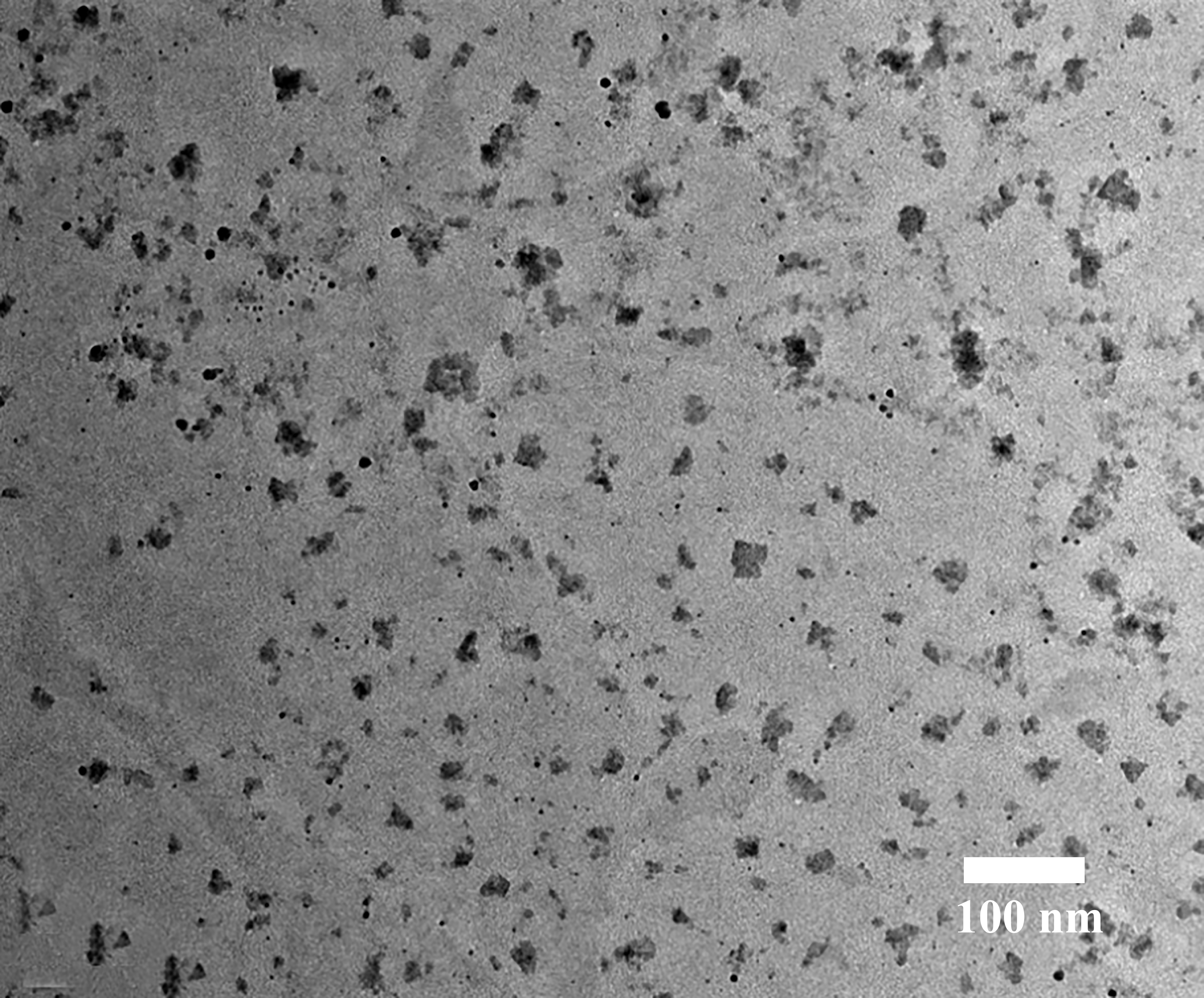


**Figure A in S1 File. TEM image of tiny GO sheets and dots**

Tiny GO sheets were prepared through photo-Fenton reaction of GO.





**Figure B in S1 File.** **XRD patterns of GO sheets and GQDs**

| Sample | Integrated emission intensity (I) | Abs. At 340 nm (A) | Refractive index of solvent (n) | Quantum yield  (Q) |
| --- | --- | --- | --- | --- |
| Quinine sulfate | 340312.957 | 0.02 | 1.33 | 0.54 |
| GQDs | 387614.334 | 0.05 | 1.33 | 0.246 |

**Table A in S1 File. Quantum yield of GQDs using quinine sulfate as a reference**

Quinine sulfate in 0.1 M H_2_SO_4_ (QY = 0.54) was chosen as a standard with GQDs. The quantum yields of GQDs (in water) were calculated according to the formula:Φ=Φs(I/Is)(A/As)(ns/n). Where Φ is the quantum yield, I is the measured integrated emission intensity, n is the refractive index of the solvent (1.33 for water), and A is the optical density. The subscript “s” refers to the reference standard with known quantum yield. To minimize reabsorption effects, absorbencies in the 10 mm fluorescence cuvette were kept under 0.1 at 340 nm.

**
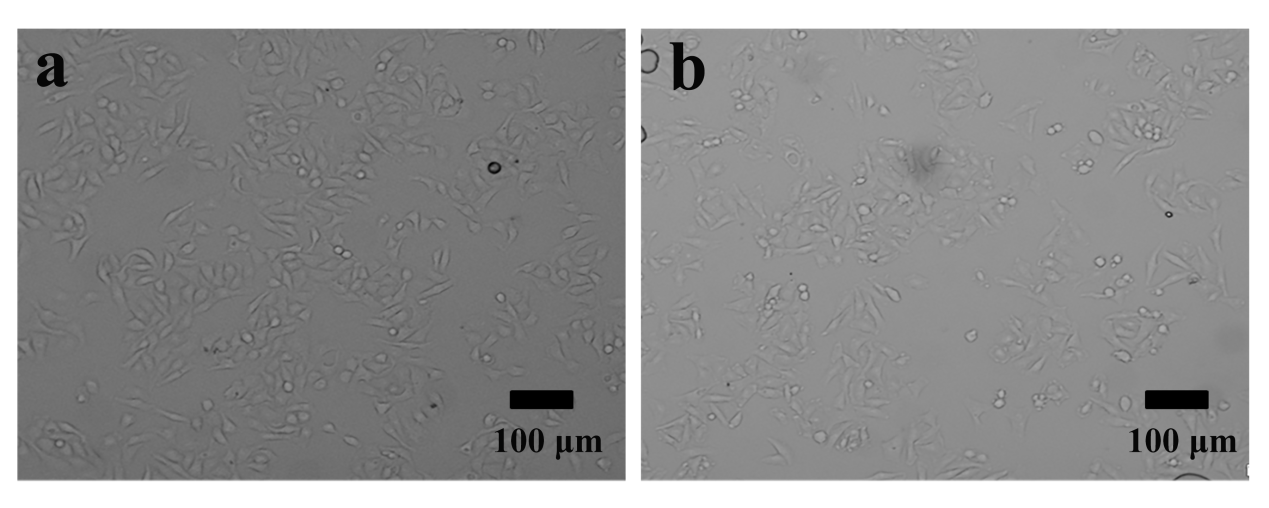
Figure C in S1 File.** **Optical microscopy images: a) the control, b) GQDs-treated cells**

HeLa cells optical microscopy images were recorded after treated with 0 and 100 μg/mL GQDs for 24 h.
